# Structural Phenomena in a Vesicle Membrane Obtained through an Evolution Experiment: A Study Based on MD Simulations

**DOI:** 10.3390/life13081735

**Published:** 2023-08-12

**Authors:** María J. Dávila, Christian Mayer

**Affiliations:** Institute of Physical Chemistry, CENIDE, University of Duisburg-Essen, 45141 Essen, Germany; christian.mayer@uni-due.de

**Keywords:** origin of life, molecular dynamics, peptide aggregation

## Abstract

The chemical evolution of biomolecules was clearly affected by the overall extreme environmental conditions found on Early Earth. Periodic temperature changes inside the Earth’s crust may have played a role in the emergence and survival of functional peptides embedded in vesicular compartments. In this study, all-atom molecular dynamic (MD) simulations were used to elucidate the effect of temperature on the properties of functionalized vesicle membranes. A plausible prebiotic system was selected, constituted by a model membrane bilayer from an equimolar mixture of long-chain fatty acids and fatty amines, and an octapeptide, KSPFPFAA, previously identified as an optimized functional peptide in an evolution experiment. This peptide tends to form the largest spontaneous aggregates at higher temperatures, thereby enhancing the pore-formation process and the eventual transfer of essential molecules in a prebiotic scenario. The analyses also suggest that peptide–amphiphile interactions affect the structural properties of the membrane, with a significant increase in the degree of interdigitation at the lowest temperatures under study.

## 1. Introduction

Given the fact that it is impossible to precisely determine when the transition from prebiotic chemistry to the first forms of cellular life occurred on Earth, it is only possible to estimate the emergence of the first living entities 4.1 and 4.2 Gyr ago constraining geological evidence [1]. It is assumed that the living organisms currently known would not exist without the simultaneous availability of liquid water, as well as soluble bioactive elements and an external energy source [2,3]. Amphiphilic molecules with the ability to generate spontaneous vesicular membranes and evolve into self-replicating systems as precursors of the first cells were probably sourced from extraterrestrial material or synthesized on Early Earth via Fischer–Tropsch-type reactions [4,5,6]. However, it seems clear that prebiotic amphiphilic compounds would have been simpler molecules than the double-chained amphiphiles forming modern cells, since the membranes formed by single-chain molecules, such as fatty acids, typically display relatively high permeability to polar and charged small molecules, as well as a high capacity for growth through the incorporation of external amphiphilic molecules [7,8,9]. The composition of protocell membranes must have been heterogeneous, incorporating not only fatty acids but also other components present in prebiotic conditions, such as fatty alcohols and glycerol monoesters [10,11,12], or alkanes and other hydrocarbons like polycyclic aromatic hydrocarbons [13]. Presumably, they allowed increased physical [11] or thermal stability [14] and even increased permeability to polar nutrients into protocells [15,16].

Similar to recent biological membranes, with their typical protein and lipid compositions, primitive cell membranes may have also contained peptides [7]. They could have been formed through condensation reactions [17,18] of amino acids sourced, as with other biomonomers, from exogenous or endogenous pathways [19]. Their formation possibly involved polymerization reactions with the continuous removal of water at high temperatures or with condensing agents, such as polyphosphates or carbonyl sulfide [20,21,22]. Simple substances, such as CO_2_, sulfite/sulfate, and ammonium, may also have been converted into peptides via Sammox (sulfite/sulfate reduction coupled to anaerobic ammonium oxidation)-driven chemical reaction networks [23]. In addition, it has been postulated that amino acid-like building blocks can also form proto-peptides, such as oligoesters [24] or depsipeptides, which can be synthesized via ester amide exchange reactions, as possible molecular ancestors of modern proteins [25]. Short peptides embedded in vesicular structures formed by simple amphiphilic molecules may have enabled the emergence of early life forms from non-living matter in the first stages of evolution. Bilayer membranes may have provided both protection for and the chemical selection of the peptides accumulated in their interior. In turn, the embedded peptides may have also stabilized the vesicular structures, thereby increasing their survivability in harsh primordial environments [26]. Through non-covalent interactions between amphiphiles and peptides, primitive ion channels may have also formed to stabilize the first cell-like structures by avoiding osmotic imbalances and to create paths for the selective permeation of crucial nutrients and ions [26,27,28].

Early cellular membranes may have emerged and survived harsh temperatures, pressure, pH, or ionic-strength gradients, which presumably occurred in diverse geological environments, such as pumice rafts, volcanic splash pools, submarine hydrothermal vents, and subaerial springs [29,30]. A wide variety of thermotropic phases may have appeared in amphiphilic membranes under these environmental conditions, thereby affecting the stability and functionality of the protocells.

In this article, we describe a theoretical study using all-atom molecular dynamics (MD) to investigate the influence of periodical temperature changes in membranous prebiotic systems with embedded short peptides. For this purpose, temperature changes from 358 K to 323 K were established, based on the hypothesis postulated by Schreiber et al., as plausible prebiotic scenarios for CO_2_-driven cold-water geysers [31]. In particular, a vesicular system constituted by the H-Lys-Ser-Pro-Phe-Pro-Phe-Ala-Ala-OH (KSPFPFAA) peptide, previously identified in an evolution experiment [26] and embedded in octadecylammonium (ODAP)/octadecanoic acid (STEP) (1:1) bilayer membranes [32], was used to achieve a better understanding the influence of temperature changes on the spontaneous peptide aggregation mechanism, as well as on the stability and structural properties of a plausible prebiotic vesicular system.

## 2. Computational Methods

All molecular dynamic simulations were performed using GROMACS version 2020, with periodic boundary conditions applied in all directions [33,34]. Each system was first energy-minimized via the steepest descent algorithm and then equilibrated for 500 ps and 40 ns in the canonical (NVT) and isothermal–isobaric (NPT) ensemble, respectively. The temperature was kept constant using the Berendsen thermostat method [35], with a coupling time constant of 0.1 ps. The pressure in the simulation systems was maintained at 1 bar using the Berendsen weak-coupling algorithm [35], with a coupling time constant of 1.0 ps. Production dynamics were examined in the isothermal–isobaric (NPT) ensemble over a temperature range of 323 K to 353 K. The temperatures were controlled by using a velocity-rescaling thermostat [36], with a coupling time constant of 0.1 ps. A pressure of 1 bar was controlled using a Parrinello–Rahman barostat [37], with a coupling time constant of 2 ps. The LINCS algorithm [38] was used to constrain all bond lengths involving hydrogen atoms in amphiphilic molecules and octapeptides. Bond lengths in water molecules were constrained using the SETTLE algorithm [39]. Coulomb interactions were calculated using the particle mesh Ewald method [40] with a real-space cutoff of 12 Å, whereas the Van der Waals interactions were treated with a cutoff at 12 Å and a switch function starting at 10 Å. The CHARMM36 force-field [41] parameters were applied for amphiphilic molecules, and the TIP3P model [42] was applied for water.

The amphiphilic bilayer was composed of 366 pairs of octadecylammonium/octadecanoic acid molecules and solvated by 15,680 water molecules. To this system, sixteen KSPFPFAA peptides were inserted perpendicular to the membrane *xy*-plane in an antiparallel arrangement at a distance of 2.8 nm, which prevented prior interaction between adjacent peptides [43]. The peptide conformation used in the simulation systems was obtained as previously reported by Dávila et al. [44], which is the most representative conformation obtained in a clustering process after the peptide simulation in an octane–water system. All bilayer systems were neutralized with sodium and chloride counterions wherever necessary.

A combination of GROMACS [33], MEMBPLUGIN [45], and g_lomepro [46] tools were used to analyze the trajectories. All figures depicting molecules throughout the paper were generated with Visual Molecular Dynamics (VMD) software [47].

## 3. Results and Discussion

Molecular dynamic simulations were used to investigate the effect of temperature on several physical properties of the membrane at molecular resolution. To characterize the amphiphilic molecular pair (octadecylammonium and octadecanoic acid)–peptide (KSPFPFAA) system, the area per amphiphile pair, bilayer thickness, curvature, interdigitation degree, and density, as well as carbon order parameters and diffusion, were calculated.

**Figure 1:**
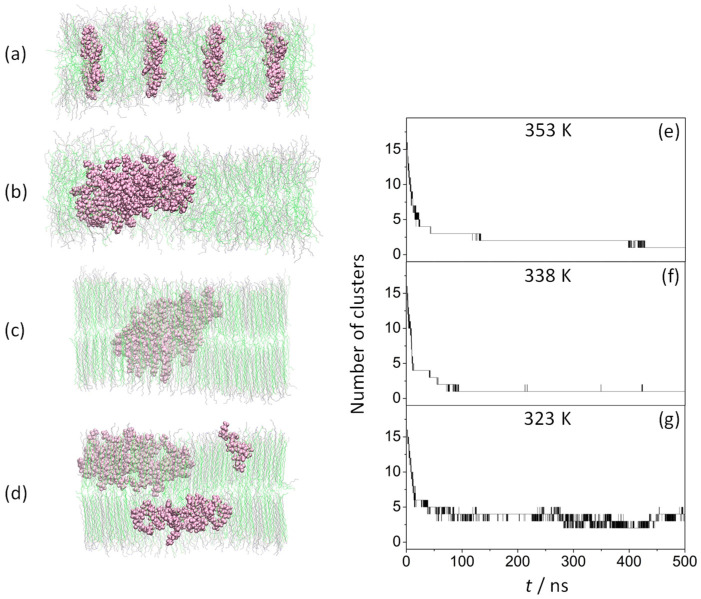
Effect of temperature on the peptide aggregation process. Left: side view of (ODAP—STEP) bilayer systems with peptides at 0 ns (**a**) and 500 ns at 353 K (**b**), 338 K (**c**), and 323 K (**d**); KSPFPFAA peptides are shown in mauve, while ODAP and STEP are in gray and green, respectively. Right: number of the clusters as a function of time formed during the 500 ns MD simulation with cutoff 0.6 nm at 353 K (**e**) (previously reported by the authors in Ref. [44]), 338 K (**f**), and 323 (**g**).

Sixteen KSPFPFAA peptides were inserted in an antiparallel arrangement, perpendicular to the membrane surface, as described in previously published research [44] (Figure 1a), followed by 500 ns MD simulations performed at 353 K (Figure 1b), 338 K (Figure 1c), and 323 K (Figure 1d).

Due to the large disorder of the amphiphilic membrane at 353 K, a rapid aggregation of the peptides was observed, resulting in the formation of a single cluster at 428 ns (these results were previously reported in Ref. [44]) (Figure 1e). At 338 K, most of the amphiphile chains were more tightly packed. However, as will be discussed in subsequent sections, disordered amphiphile chains were also found, both factors likely responsible for the very rapid aggregation. Thus, the formation of the first peptide dimer is observed within the first 2 ns, ending the aggregation process at 89 ns by stabilizing a single cluster (Figure 1f). However, at 323 K (Figure 1g), only a stable 10-peptide cluster formed at 86 ns and above, with no peptide exchange between clusters over the simulation period of 500 ns, due to the high degree of order in the alkyl chains of the amphiphiles.

**Figure 2:**
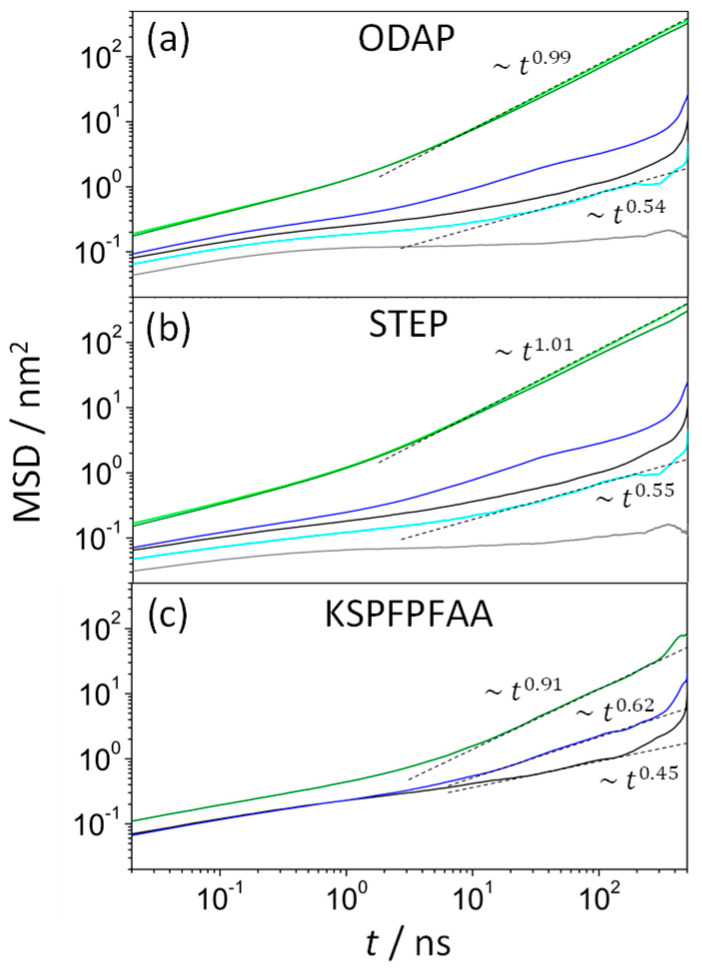
Lateral mean square displacements as a function of time for ODAP (**a**), STEP (**b**), and KSPFPFAA (**c**) in an ODAP–STEP–KSPFPFAA system at 353 K (green), 338 K (blue), and 323 (black), and in ODAP–STEP bilayers at 353 K (light green), 338 K (light blue), and 323 (grey).

A diffusion analysis of the components of the systems was also carried out (Figure 2). The lateral mean square displacement (MSD) is defined by

$$\langle {∆r\;\left(t\right)}^{2}\rangle \;=\frac{1}{N}\;\sum_{i=1}^{N}{\left\{r_{i}\left(t\right)-r_{i}\left(0\right)\right\}}^{2}$$

where $r_{i}$ is the (x,y) position of the center of mass of the molecule *i*, and *N* is the number of molecules.

In normal Brownian diffusion, characterized by $\langle {∆r\;\left(t\right)}^{2}\rangle \;\sim \;t$ for long times, the lateral diffusion coefficient, $D_{\mathrm{lat}}$, can be estimated by using the Einstein equation.

$$D_{\mathrm{lat}}=\frac{1}{4}\;\underset{t\to \infty }{\lim}\frac{\langle {∆r\;(t)}^{2}\rangle \;}{t}$$

At 353 K, the values of the lateral diffusion coefficients were 1.89 × 10^−6^ cm^2^/s (ODAP, Figure 2a) and 1.95 × 10^−6^ cm^2^/s (STEP, Figure 2b) in ODAP–STEP bilayers. These values for $D_{\mathrm{lat}}$ indicate that both amphiphilic molecules diffuse much faster than most phospholipids with $D_{\mathrm{lat}}$ values of the order of 10^−8^ cm^2^/s [48]. On the other hand, $D_{\mathrm{lat}}$ values obtained in this work at 353 K are consistent with those reported in the literature for other fatty acids, such as decanoic acid in vesicles containing its deprotonated anion with $D_{\mathrm{lat}}$ = 9 × 10^−6^ cm^2^/s [49]. In the peptide-containing systems at 353 K, a slight decrease in the lateral diffusion coefficients was observed compared with the previous system, with the values of 1.64 × 10^−6^ cm^2^/s (ODAP, Figure 2a) and 1.40 × 10^−6^ cm^2^/s (STEP, Figure 2b). The KSPFPFAA peptides diffused even more slowly than ODAP and STEP, with $D_{\mathrm{lat}}$ values of 2.68 × 10^−7^ cm^2^/s (Figure 2c; it is worth noting that a slight deviation from the linear diffusion regime was observed for the MSD time variation). Thus, it can be assumed that, in the liquid-disordered (Ld) phase, KSPFPFAA peptides slow down the lateral diffusion of both amphiphiles (the structural properties analyzed in this section will confirm the existence of the Ld phase at 353 K). As the temperature of ODAP–STEP bilayers decreased, the diffusion of the amphiphiles slowed down and started to exhibit anomalous behavior. The subdiffusive regime of both amphiphiles was then characterized by ${\langle {∆r\;\left(t\right)}^{2}\rangle \;\sim \;t}^{\alpha }$, with anomalous diffusion exponents *α* between 0.55 (338 K) and 0.15 (323 K) for long diffusion times, strongly decreasing with decreasing temperature. However, in peptide-containing bilayer systems between 338 K and 323 K, *α* varied only slightly with the temperature, being 0.5 for ODAP and STEP amphiphiles but progressively decreasing to 0.62 (338 K) and 0.44 (323 K) for KSPFPFAA peptides. As expected, these results show that amphiphiles diffuse more slowly in the gel (S) phase than in the liquid phase (The S phase was confirmed in the structural properties reported in this work). The temperature-related decrease in amphiphile mobility in the peptide-containing S phase was obviously less pronounced than that in the pure S phase, probably caused by a disorder in the alkyl chains of the amphiphiles generated by the peptide.

**Figure 3:**
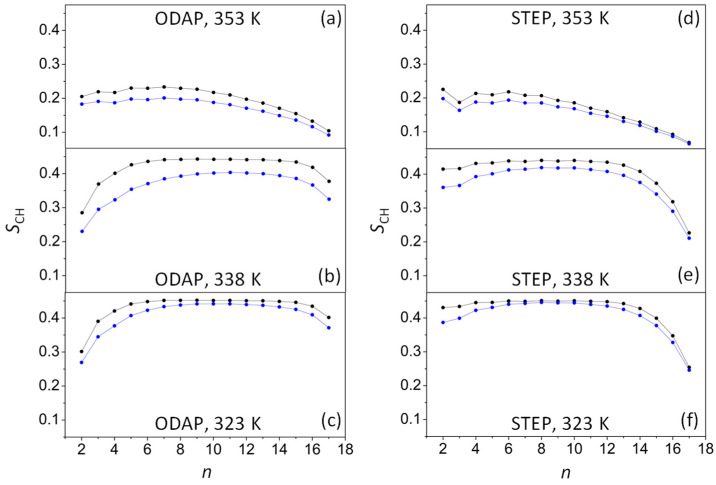
Order parameters (*S*_CH_) for ODAP at 353 K (**a**), 338 K (**b**) and 323 K (**c**), and STEP at 353 K (**d**), 338 K (**e**), and 323 K (**f**) in an ODAP–STEP bilayer (black lines) and in an ODAP–STEP bilayer containing KSPFPFAA (blue lines).

The order parameter (*S*_CH_) is widely used to characterize the ordering of the bilayer’s hydrophobic region (Figure 3). *S*_CH_ can be estimated for every CH_2_ group in the chains according to the following equation:

$$S_{\mathrm{CH}}=\frac{1}{2}\langle 3{\;\cos}^{2}\;\theta -\;1\rangle$$

where θ is the angle between the CH bond and the bilayer normal (the angle bracket designates the ensemble average) [50]. Therefore, *S*_CH_ may have values ranging from −0.5 to 1, denoting amphiphile molecules strictly oriented perpendicular or parallel to the *z*-axis, respectively.

At 353 K, *S*_CH_ values ranged from 0.233 and 0.201 at the top of amphiphilic chains to 0.068 and 0.064 at the terminal methyl groups in ODAP–STEP bilayers and in the ODAP–STEP–KSPFPFAA system, respectively (Figure 3a,d). These *S*_CH_ values suggest the existence of an Ld phase at 353 K for both systems. The significant decrease in these values in the presence of the integrated peptide suggests a substantial increase in the disorder of the alkyl chains. As the temperature dropped to 338 K in the ODAP–STEP bilayer, *S*_CH_ values increased, ranging from 0.443 to 0.226 (Figure 3b,e). At 323 K, these values were even higher, ranging from 0.452 to 0.254 (Figure 3c,f). Thus, it can be assumed that, at 338 K as well as at 323 K, the membrane was in the S phase, exhibiting further gel-like ordering in the amphiphilic alkyl chains as the temperature decreased, similar to a phospholipid bilayer [51]. In peptide-containing bilayer systems at 338 K (Figure 3b,e), *S*_CH_ values ranged from 0.419 to 0.211, while at 323 K (Figure 3c,f), these values ranged from 0.446 to 0.246. Therefore, at 338 K, a marked reduction was observed in the *S*_CH_ values of alkyl chains compared with those in the peptide-free ODAP–STEP bilayer, which may be due to the increased disorder of the amphiphile molecules closest to the peptides [52,53].

The degree of interdigitation between the alkyl chains of opposite leaflets in each system was also determined by computing the mass distribution overlap of the two leaflets as follows [45]:

$$I_{\rho }^{2}=4\int \frac{\rho_{a}\left(z\right)\rho_{b}\left(z\right)}{{\left(\rho_{a}\left(z\right)+\rho_{a}\left(z\right)\right)}^{2}}\;dz$$

where $\rho_{a}$ and $\rho_{b}$ represent the mass density profiles for each of the leaflets. The fraction of mass overlap, $I_{\rho }$, varies between 0 and 1, meaning no mass overlap or full mass overlap, respectively.

In the ODAP–STEP bilayer, the mass density overlap was significantly higher at 358 K than at 323 K and 338 K, probably caused by a decrease in membrane packing, leading to an increase in the interaction region between the amphiphile molecules [54]. In the peptide-containing system at 353 K, a slight increase in the interaction between the two leaflets was found in relation to the peptide-free bilayer. However, at 338 K and 323 K, $I_{\rho }$ values sharply increased for the ODAP—STEP—KSPFPFAA system, which may be a result of increased amphiphiles disorder connected to the formation of peptide aggregates (Table 1).

**Figure 4:**
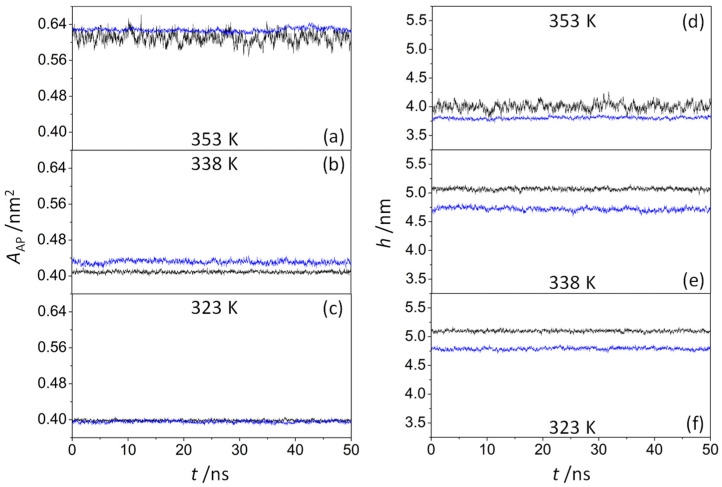
Time-dependent area per amphiphile pair *A*_AP_ (**left**) and membrane thickness *h* (**right**) of a pure ODAP–STEP bilayer (black lines) and an ODAP–STEP bilayer containing KSPFPFAA peptides (blue lines) at 353 K (**a**,**d**); 338 K (**b**,**e**); and 323 K (**c**,**f**).

The average area per amphiphile pair (*A*_AP_) depends on the hydrophilic attraction of the head groups of amphiphilic molecules as well as on the steric repulsion of the tails of non-polar hydrocarbons. It can be calculated for a pure amphiphilic model system as follows:

$$A_{\mathrm{AP}}=A_{\mathrm{xy}}/N$$

where *A*_xy_ is the sectional area of the system along the bilayer *xy*-plane, and *N* is the number of amphiphile pairs in a single leaflet. However, given the difficulty to obtain this value for peptide-containing systems, their areas were analyzed using the method developed by Gapsys et al. [46], which is based on the GridMAT-MD algorithm [55]. Figure 4a–c show that the time evolution of the area per amphiphile pair did not change significantly during the last 50 ns trajectory, concluding that all the systems reached a steady state. As expected, the average area per amphiphile pair increased as the temperature increased, varying from 0.398 nm^2^ (323 K) to 0.610 nm^2^ (353 K) in the pure amphiphilic systems and from 0.395 nm^2^ (323 K) to 0.628 nm^2^ (353 K) in octapeptide-containing systems. These results are consistent with those reported by Tian and Chiu [56] for ion-pair amphiphile membranes, decreasing the average area of the octadecyltrimethylammonium–octadecylsulfate complex from 0.556 nm^2^ (366 K) to 0.441 nm^2^ (288 K), thus confirming the transition from the liquid-disordered phase to the gel phase (solid-like phase). At 353 K (Figure 4a), the ODAP–STEP bilayers were obviously in the Ld phase, with the *A*_AP_ values fluctuating between 0.568 nm^2^ and 0.661 nm^2^. However, the *A*_AP_ values were more stable in peptide-containing systems, varying from 0.617 nm^2^ to 0.645 nm^2^. Hence, KSPFPFAA peptides integrated into the membrane may have a stabilizing effect on the structure of the Ld phase bilayer. At 323 K (Figure 4c), no significant changes in the area per amphiphile pair were observed when peptides were incorporated. In contrast, an increase in this property was observed at 338 K (Figure 4b). Here, the average area per amphiphile pair increased from 0.409 nm^2^ to 0.431 nm^2^ after the integration of the peptide, mainly caused by an increase in the disorder of alkyl chains and with the coexistence of Ld and S phases.

**Figure 5:**
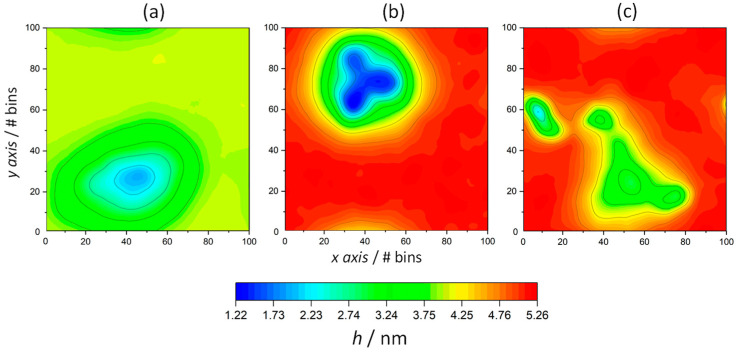
The bilayer thickness profile of KSPFPFAA-containing ODAP–STEP bilayers at 353 K (**a**), 338 K (**b**), and 323 K (**c**). Red regions represent high thickness and blue regions represent low thickness.

The bilayer thickness over both leaflets was calculated by means of the g_lomepro tool [46]. Hence, the distances between the nitrogen atoms of octadecylammonium located in the opposite leaflet layers were considered as references, using a grid of 100 × 100 points to define their location. As shown in Figure 4d,e, the average membrane thickness exhibits the opposite tendency of the area per lipid. With increasing temperature, the membrane thickness decreased in all systems, ranging from 5.10 nm (323 K) to 4.00 nm (353 K) in ODAP–STEP bilayers, and from 4.79 nm (323 K) to 3.80 nm (353 K) in peptide-containing bilayers. At 323 K (Figure 4f) and 338 K (Figure 4e), smaller membrane thicknesses were observed after peptide insertion, leading to values of 0.31 nm and 0.35 nm, respectively. A comparison of the 2D thickness maps reveals a pronounced peptide-induced bilayer thinning down to 1.22 nm at 338 K (Figure 5b), whereas at 323 K, the minimum local bilayer thickness was 2.21 nm (Figure 5c). At 353 K (Figure 4d), ODAP–STEP bilayers were in the Ld phase, with a fully disordered structure due to the thermal fluctuations, thus exhibiting a drastic decrease in the average membrane thickness compared with S-phase bilayers, which can be attributed to more disordered hydrophobic chains in the Ld phase. The corresponding peptide-induced membrane thickness reduction of 0.20 nm was not as significant as that in S-phase bilayers. Moreover, the time evolution of the membrane thickness in the peptide-containing system shows fluctuations in the range of 0.17 nm at 353 K, in contrast to 0.48 nm for the pure system. The addition of the peptide likely provides a certain stability despite the thickness reduction. At this temperature, a peptide cluster forms in both monolayers, resulting in a local bilayer thinning of up to 1.95 nm (Figure 5a). This effect, which was already observed at 338 K, can be attributed to a deformation of the membrane in response to a negative hydrophobic mismatch [57].

**Figure 6:**
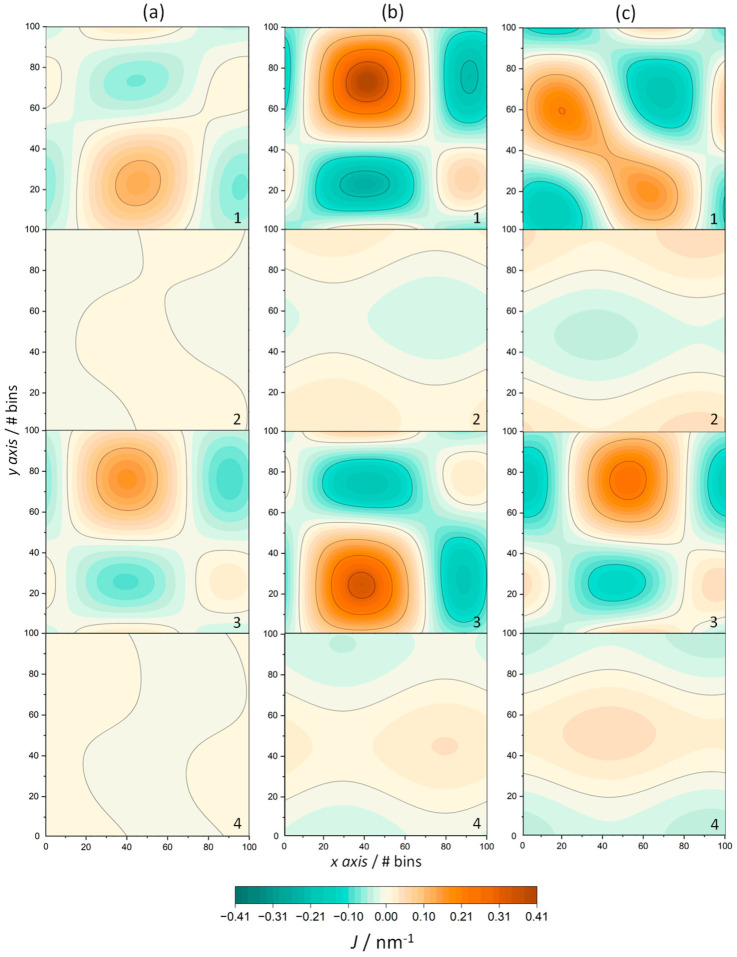
Mean curvature, *J*, of ODAP–STEP bilayer (2, top leaflet; 4, bottom leaflet) and peptide-containing ODAP–STEP bilayers (1, top leaflet; 3, bottom leaflet) at 353 K (**a**), 338 K (**b**), an 323 K (**c**). Orange regions represent positive membrane curvatures and turquoise regions represent negative curvatures.

In addition, the mean curvature *J* was calculated using the g_lomepro tool [46] for pure ODAP—STEP and peptide-containing ODAP—STEP bilayer systems, at 353 K, 338 K, and 323 K (Figure 6). The mean curvature is defined as follows:

$$J=\left(EN+GL-2FM\right)/\left(2{\left(EG-F\right)}^{2}\right)$$

The coefficients of the first fundamental form *E*, *F*, and *G* are $E=S_{x}\cdot S_{x}$, $F=S_{x}\cdot S_{y}$, and $G=S_{y}\cdot S_{y}$, with *S_x_* and *S_y_* vectors for every grid cell (*x*, *y*) for both leaflets, $S_{x}=\partial S/\partial x$ and $S_{y}=\partial S/\partial y$. (The coordinates over a surface *S* = *S(x*, *y)* are obtained by assigning grid points to the corresponding lipid coordinates along the normal of the bilayer.) The unit normal to the surface at every grid point, calculated as $N=(S_{x}\times S_{y})/\left\|S_{x}\times S_{y}\right\|$, was used to estimate the coefficients of the second fundamental form, $L=S_{xx}\cdot N$, $M=S_{xy}\cdot N$, and $N=S_{yy}\cdot N$, with ${S_{xx}=\partial^{2}S/\partial x}^{2}$, ${S_{yy}=\partial^{2}S/\partial y}^{2}$, and $S_{xy}=\partial^{2}S/\partial x\partial y$.

A positive mean curvature is assigned to the surface bending toward the bilayer center, while a negative mean curvature is assigned to the surface bending away from the bilayer center, as described in Ref. [46] (Figure 6). The small mean curvatures of the ODAP—STEP bilayers at 353 K (Figure 6a), ranging from 0.013 nm^−1^ to −0.011 nm^−1^, can be attributed to thermal fluctuations. However, the mean curvature slightly increased in the gel phase, ranging from 0.035 nm^−1^ to −0.035 nm^−1^ at 338K (Figure 6b), and from 0.045 nm^−1^ to −0.045 nm^−1^ at 323 K (Figure 6c), probably due to a partially interdigitated gel state (top and bottom leaflets are shown in Figure 6(2) and Figure 6(4), respectively). In the peptide-containing ODAP—STEP bilayer system, large fluctuations in the membrane surface were observed. In addition, peptide aggregation led to a significant positive mean curvature. At 353 K, as in the peptide-free membrane, the mean curvatures were considerably smaller than those at 323 K and 338 K, ranging from 0.127 nm^−1^ to −0.072 nm^−1^ (top leaflet) and from 0.161 nm^−1^ to −0.097 nm^−1^ (bottom leaflet). This is a consequence of both the existence of large thermal fluctuations and, as evident in the above properties, a conformational disorder with the membrane in the liquid-disordered phase. The positive mean curvature shown in Figure 6b at 338 K in the top and bottom leaflet, with values up to 0.408 nm^−1^ and 0.342 nm^−1^, respectively, was significantly more pronounced than that at 323 K (Figure 6c), with mean curvatures up to 0.206 nm^−1^ (top leaflet) and 0.244 nm^−1^ (bottom leaflet). This pronounced difference may be due to the formation of larger clusters at 338 K as previously discussed, which leads to a larger curvature on the membrane surface.

**Figure 7:**
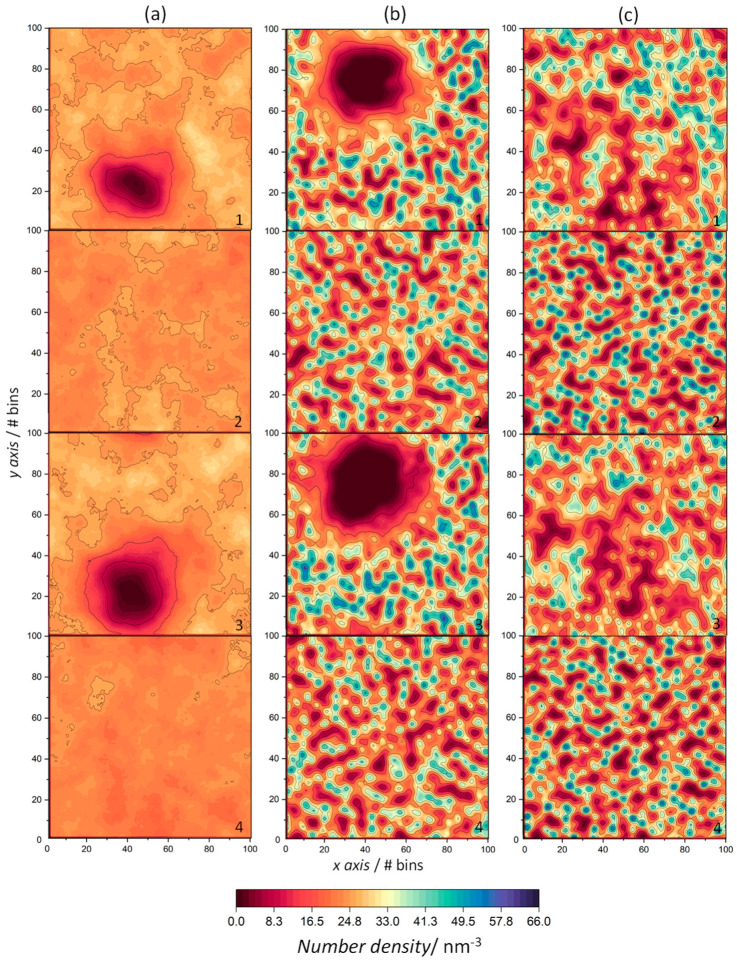
The 2D number density maps of ODAP–STEP bilayer (2, ODAP; 4, STEP) and peptide-containing ODAP–STEP bilayers (1, ODAP; 3, STEP) at 353 K (**a**), 338 K (**b**), and 323 K (**c**). Dark red regions represent low number densities and dark blue regions represent high number densities.

In order to gain additional insight into the lateral distribution of amphiphilic molecules within the membrane, 2D number density maps of each component in the *xy*-plane were plotted (Figure 7).

At 353 K, Figure 7a(2,4) reveal a uniform distribution of both amphiphiles in the ODAP–STEP bilayer without aggregate formation, with maximum density values of 29.3 nm^−3^ and 27.1 nm^−3^. As indicated by the properties mentioned above, a high mobility of lipid molecules was observed at 353 K. This is reflected in the density maps that display a very homogeneous distribution of the lipids. ODAP and STEP densities increased with decreasing temperature, with maxima of 55.6 nm^−3^ (ODAP, Figure 7b(2)) and 53.2 nm^−3^ at 338 K (STEP, Figure 7b(4)), and 65.6 nm^−3^ (ODAP, Figure 7c(2)) and 61.0 nm^−3^ (STEP, Figure 7c(4)) at 323 K. At the lowest temperature in the S phase under study, the amphiphiles pack more closely, which is reflected by an increase in number density values as well as by an increased membrane thickness. In peptide-containing membranes (Figure 7a(1,3)–c(1,3)), a pronounced decrease in number densities was observed in the amphiphiles close to peptide aggregates, which was more evident at 353 K and 338 K. In addition, an increase in disorder occurred in these neighboring amphiphiles near the peptide aggregates at 338 K and 323 K. This effect was particularly evident at 338 K with a more uniform lateral distribution of the amphiphiles.

**Figure 8:**
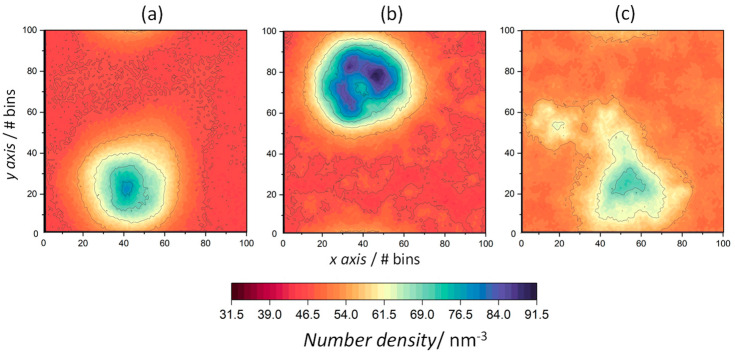
The 2D number density maps for water in peptide-containing ODAP–STEP bilayers at 353 K (**a**), 338 K (**b**), and 323 K (**c**). Dark red regions represent low number densities and dark blue regions represent high number densities.

The density distribution of the water molecules was also mapped onto the *xy*-plane to quantify the water penetration into membrane structures (Figure 8). During the peptide aggregation process at 353 K, pores are formed [44]. Consequently, water molecules penetrate the membrane, which leads to a water number density maximum of 79.6 nm^−3^. At 338 K and 323 K, the number density maps also reveal the penetration of water molecules into the bilayer through peptide aggregates, with density maxima of 91.5 nm^−3^ and 72.4 nm^−3^. Such differences in densities may be due to the fact that, at 338 K, the fluctuations of the amphiphiles surrounding the peptides increase, with a consequent increase in the disorder of the lamellar structure of the S phase, leading to the increased permeation of water molecules through the peptide aggregates.

## 4. Conclusions

Under harsh temperature conditions in prebiotic environments, early cellular membranes may have developed primitive functions, leading to their long-term survival. Similar to current biological membranes, they would have existed predominantly in a liquid-crystalline phase with the occasional coexistence of gel-like regions. In order to gain a better understanding of the structure and dynamics of a plausible prebiotic system as a function of temperature, all-atom molecular dynamic simulations were carried out. In the bilayer formed via a combination of two amphiphiles, a decrease in temperature leads to a closer packing of both components, resulting in a decreased amphiphile mobility as well as an increase in bilayer thickness. However, close to the liquid-gel phase transition temperature, both phases coexist in the peptide-containing membrane, leading to local bilayer thinning, an increase in the amphiphile diffusion in the immediate vicinity of peptide aggregates, and an increase in the density of water molecules penetrating the bilayer structure. Consequently, this study may be useful to further explore the stabilization and functionalization mechanisms of protocells in Earth’s crust.

## Figures and Tables

**Table 1:** Degree of interdigitation (*I_ρ_*), the average area per amphiphile pair (*A*_AP_), and bilayer thickness (*h*) obtained for the last 50 ns of the MD trajectory. The standard deviations are reported in parentheses.

System	*T*/K	*I_ρ_*	*A*_AP/_nm^2^	*h*/nm
**ODAP—STEP**	353	0.56 (0.06)	0.610 (0.013)	4.00 (0.07)
	338	0.20 (0.02)	0.409 (0.003)	5.07 (0.03)
	323	0.19 (0.02)	0.398 (0.002)	5.10 (0.02)
**ODAP—STEP—KSPFPFAA**	353	0.60 (0.07)	0.628 (0.004)	3.80 (0.02)
	338	0.31 (0.02)	0.431 (0.004)	4.72 (0.03)
	323	0.30 (0.02)	0.395 (0.003)	4.79 (0.02)

## Data Availability

All data generated during this study are available from the corresponding author upon request.
